# Supplementary material for: Spatial arrangement of the whiskers of harbor seals (Phoca vitulina) compared with whisker arrangements of house mice (Mus musculus) and brown rats (Rattus norvegicus)
Source: J Exp Biol. 2024 Nov 27;227(22):jeb247545. doi: 10.1242/jeb.247545 (PMC11634035; doi:10.1242/jeb.247545)
Supplement: Supplementary information [file jexbio-227-247545-s1.pdf]

Table S1. Best fit equations for radial basepoint coordinate and angles of emergence by individual animal, along with combined equations across animals. *Abbreviations:* Prm: Parameter; (n): number of whiskers.

	Prm	Animal (n)	Coefficients			95% Confidence Intervals			Adj. R^2
			c_1	c_2	c_3	c_1	c_2	c_3	
1	r_{bp}	Seal 1 (56)	N/A	-0.42203	36.525	N/A	[-0.4729, -0.3711]	[35.5827, 37.4682]	0.833
		Seal 2 (62)	0.003283	-0.53157	35.899	[4.970e-04, 0.0060702]	[-0.5945, -0.4687]	[34.6273, 37.1707]	0.838
		Seal 3 (53)	0.004162	-0.61645	35.195	[0.001343, 0.006980]	[-0.6877, -0.5452]	[33.8951, 36.4957]	0.882
		Combined (171)	N/A	-0.4905	36.489	N/A	[-0.5236, -0.4574]	[35.8804, 37.0967]	0.846

Final equation for radial basepoint coordinate: $r_{bp} = c_1\theta_{bp}^2 + c_2\theta_{bp} + c_3$, where r_{bp} is in millimeters and θ_{bp} is in degrees.

	Prm	Animal (n)	c_1	c_2	c_3	c_4	c_1	c_2	c_3	c_4	Adj. R^2
2	θ_w	Seal 1 (56)	N/A	-0.972	1.815	133.02	N/A	[-1.1597, -0.7849]	[1.6454, 1.9849]	[129.9012, 136.1351]	0.903
		Seal 2 (62)	N/A	-1.141	1.647	138.42	N/A	[-1.3181, -0.9631]	[1.4731, 1.8199]	[135.3208, 141.5222]	0.889
		Seal 3 (53)	0.0504	-0.749	1.856	133.85	[0.0394, 0.0615]	[-0.9656, -0.5319]	[1.6882, 2.0236]	[129.8400, 137.8607]	0.923
		Combined (171)	N/A	-1.100	1.770	138.42	N/A	[-1.2346, -0.9655]	[1.6481, 1.8927]	[136.1603, 140.6733]	0.854

Final equation for azimuthal angle of emergence: $\theta_w = c_1\phi_{bp}^2 + c_2\phi_{bp} + c_3\theta_{bp} + c_4$, where θ_w, ϕ_{bp} and θ_{bp} are in degrees.

	Prm	Animal (n)	c_1	c_2	c_3	c_4	c_1	c_2	c_3	c_4	Adj. R^2
3	ϕ_w	Seal 1 (56)	N/A	1.700	N/A	-23.345	N/A	[1.5198, 1.8800]	N/A	[-26.3311, -20.3596]	0.867
		Seal 2 (62)	N/A	1.449	N/A	-21.738	N/A	[1.3460, 1.5526]	N/A	[-23.4949, -19.9809]	0.928
		Seal 3 (53)	0.0127	1.730	N/A	-19.799	[0.003515, 0.02179]	[1.5494, 1.9098]	N/A	[-22.9582, -16.6397]	0.882
		Combined (171)	N/A	1.570	N/A	-20.928	N/A	[1.4802, 1.6597]	N/A	[-22.3935, -19.4632]	0.875

Final equation for elevation angle of emergence: $\phi_w = c_1\phi_{bp}^2 + c_2\phi_{bp} + c_3\theta_{bp} + c_4$, where ϕ_w, ϕ_{bp}, and θ_{bp} are in degrees.

	Prm	Animal (n)	c_1	c_2	c_3	c_4	c_1	c_2	c_3	c_4	Adj. R^2
4	ζ_w	Seal 1 (56)	N/A	-1.350	1.573	-23.663	N/A	[-1.7195, -0.9803]	[1.2382, 1.9078]	[-29.8110, -17.5155]	0.693
		Seal 2 (62)	N/A	-1.565	1.753	-23.714	N/A	[-1.8397, -1.2893]	[1.4836, 2.0214]	[-28.5220, -18.9053]	0.818
		Seal 3 (53)	N/A	-1.732	2.308	-28.091	N/A	[-2.1166, -1.3482]	[1.9916, 2.6238]	[-34.2624, -21.9194]	0.840
		Combined (171)	N/A	-1.540	1.866	-24.447	N/A	[-1.7351, -1.3448]	[1.6882, 2.0429]	[-27.7197, -21.1746]	0.784

Final equation for twist angle of emergence: $\zeta_w = c_1\phi_{bp}^2 + c_2\phi_{bp} + c_3\theta_{bp} + c_4$, where ζ_w, ϕ_{bp}, and θ_{bp} are in degrees.
